# Toll-Like Receptor Signaling in Liver Diseases

**DOI:** 10.1155/2010/971270

**Published:** 2011-05-05

**Authors:** Gyongyi Szabo, Timothy R. Billiar, Keigo Machida, Ian Nicholas Crispe, Ekihiro Seki

**Affiliations:** ^1^Department of Medicine, University of Massachusetts Medical School, Worcester, MA 01605, USA; ^2^Department of Surgery, University of Pittsburgh Medical Center, Pittsburgh, PA 15213, USA; ^3^Department of Molecular Microbiology and Immunology, University of Southern California School of Medicine, Los Angeles, CA 90033, USA; ^4^Malari Program, Seattle Biomedical Research Institute, Seattle, WA 98109, USA; ^5^Department of Medicine, School of Medicine, University of California, San Diego, La Jolla, CA 92093, USA

*Drosophila* Toll was initially discovered as the factor responsible for determining dorsoventral polarity in *Drosophila* embryo, and the subsequent studies revealed its antifungal functions. In the late 1990s, mammalian homologs of Toll were determined as Toll-like receptors (TLRs). Early studies on TLRs attempted to identify their specific ligands, new family member of TLRs, and their intracellular signal transduction pathways. Intracellular signal transductions of TLRs share common elements with IL-1 receptor downstream intracellular signaling. Subsequent studies for TLRs have focused on infectious diseases, since TLRs recognize pathogen-associated molecular patterns (PAMPs) and induce strong responses for host defense. Recently, endogenous TLR ligands released from dying and/or damaged cells were identified. These studies have extended the idea that TLR signaling is also activated in the absence of exogenous pathogens and in general sense “danger signals” to alert the host of either exogenous or endogenous “danger.” 

Liver has a unique anatomy that is closely associated with the intestines through the portal vein and bile ducts. Even when intestinal barrier functions are intact, liver is constantly exposed to low levels of microbial products derived from commensal microflora through portal vein blood. Liver contains not only parenchymal hepatocytes, but also nonparenchymal immune cells and nonimmune cells. Hepatic immune cells include Kupffer cells (resident liver tissue macrophages), T cells, B cells, dendritic cells, NK cells, and NKT cells. These cells produce a broad array of cytokines upon activation of TLRs. nonimmune cells in the liver include endothelial cells, biliary epithelial cells, and hepatic stellate cells. Hepatic nonimmune cells also express TLRs and respond to TLR ligands to induce innate immune responses including cytokine and type I IFN production. However, the liver in normal condition prevents spontaneous inflammation induced by microbial products through TLRs due to the specific barrier functions in the liver and intestines or the “liver tolerance” regulated by intercellular and intracellular mechanisms. Upon breakdown of this regulation, inflammation is induced through innate immune responses including TLR signaling in the liver. Moreover, sterile inflammation-associated danger signals may also trigger liver inflammation through TLRs. Thus, acute and chronic liver diseases are highly associated with triggering TLR signaling by intestine-derived microbial products and sterile insult-associated products from damaged cells. 

In the present special issue of *Toll-Like Receptor Signaling in Liver Diseases*, the most recent advances in TLR research in the liver are reviewed by worldwide authorities of liver TLR research. The first part of this issue overviews TLR signaling in general. In the first paper, Drs. M. Yamamoto and K. Takeda reviews the current views of TLR signaling including their discovery of the functions of all four TLR adaptor proteins by generating knockout mice. In the second paper, Drs. A. E. Bigorgne and I. N. Crispe review the area of hepatic intercellular crosstalk mediated by TLRs. In the third paper, the research group of Drs. M. Fujimoto and T. Naka have cloned SOCS-1 and reported that SOCS-1 negatively regulates TLR signaling by SOCS-1. They summarize the negative regulation of TLRs by SOCS proteins and the previous reports studying the SOCS family in human liver disease. In the next paper, Dr. H. Tsutsui and her colleagues summarize their studies that have identified TLR-mediated IL-1*β* and IL-18 processing through activation of the inflammasome in the liver. In the fifth paper, Dr. M. Gale Jr. and his research group have uncovered the intracellular innate immune signaling against HCV infection. He and his colleague review the biological host response against HCV and how HCV escapes from RIG-I-dependent innate immune response for sustaining HCV infection. 

The next sections address specific liver functions and diseases. In the sixth paper, Dr. K. Machida summarizes his previous work studying the association between HCV and TLR4, and now his research is extending to the study of HCV-mediated tumorigenesis in which cancer stem cells could be crucial, and its possible cross-talk with alcohol. In the following paper, Drs. Y. Iimuro and J. Fujimoto review molecular mechanisms triggering liver regeneration after partial hepatectomy. TNF-*α* and IL-6 are known to trigger liver regeneration. They discuss the role of TLR/MyD88-dependent signaling upstream TNF-*α* and IL-6 in liver regeneration. They also describe the possible interaction between NF-*κ*B and JNK/c-Jun in liver regeneration. In the eighth paper, cutting-edge research on TLRs in ischemia-reperfusion injury has been done by the research group led by Drs. A. Tsung and T. Billiar. They were the first to identify that HMGB-1 released from damaged cells is an endogenous ligand for TLR4 and that HMGB-1 triggers ischemia-reperfusion liver injury through TLR4. The interaction between alcoholic liver disease and TLR signaling has been studied for more than ten years. Dr. Thurman's research group demonstrated the role of TLR4 and gut microflora in alcoholic liver disease using intragastric ethanol infusion model. Currently, Dr. Szabo's research group advances the studies in this field. In ninth paper, she and her colleagues concisely summarize the importance of TLRs and adaptor molecules in alcoholic liver disease. The importance of TLRs has been demonstrated not only in alcoholic liver disease, but also in nonalcoholic liver disease. In the tenth paper, Drs. K. Miura and D. Brenner outline the recent advance of TLRs and nonalcoholic liver disease. In the next paper, Dr. E. Seki and his colleagues have demonstrated the interaction of the TLR4 and TGF-*β* signaling pathways in hepatic stellate cells. They discuss the signaling of TLR4 and the other TLRs in the activation of hepatic stellate cells and liver fibrosis. The proportion of NK cells and NKT cells in liver is much greater than that in the other organs. These cells are major targets for TLR3 ligand poly I:C. Dr B. Gao's research group is focsing on this signaling in liver physiology and in liver disease models. In the twelfth paper, they summarize their recent findings of TLR3 signaling in liver disease. In the following paper, Dr. S. Maeda demonstrated the role of hepatocyte NF-*κ*B, JNK, and MyD88 using an animal model of liver cancer. He discusses the role of TLRs, NF-*κ*B, and JNK signaling in liver cancer. Modification of gut microflora might be a potential target for liver disease because of the unique anatomical association between the liver and intestines. In the fourteenth paper, Dr. I. Hines and his colleague review the molecular mechanisms by which gut microflora contributes to the development of liver disease. In the fifteenth paper, Drs. T. Hayashi and K. Suzuki highlight their previous work on TLR4-mediated expression of protein S and C4b binding protein in the liver. The role of TLR4 signaling in the development of insulin resistance is intensively being studied. In the last paper, Drs. J. J. Kim and D. D. Sears summarize the recent advances in the field of innate immunity and insulin resistance. 

In this special issue, we gather 16 articles highlighting our current knowledge of TLR signaling in the liver. TLR signaling induces the initial response in inflammation. This response may then induce intestinal barrier destruction and subsequent bacterial translocation as well as the release of endogenous ligands from damaged cells. These events could cause a secondary activation of TLR signaling. Thus, targeting either the initial or secondary responses induced by TLR signaling might become effective therapy for liver diseases. While we could not include discussion of the potential of TLR agonists for the therapy of liver disease in this special issue due to page limitations, this might be an attractive approach for some liver diseases, such as liver fibrosis and cancer. We trust that the articles in this special issue will help in understanding the TLR-related mechanisms underlying liver diseases and stimulate new ideas for targeting TLRs and their signaling pathways to develop new therapeutic applications for liver disease. 

*Gyongyi Szabo* *Gyongyi Szabo* *Timothy R. Billiar* *Timothy R. Billiar* *Keigo Machida* *Keigo Machida* *Ian Nicholas Crispe* *Ian Nicholas Crispe* *Ekihiro Seki* *Ekihiro Seki*

